# Supplementary figures and images for: Four different mechanisms for switching cell polarity
Source: PLoS Comput Biol. 2021 Jan 19;17(1):e1008587. doi: 10.1371/journal.pcbi.1008587 (PMC7861558; doi:10.1371/journal.pcbi.1008587)

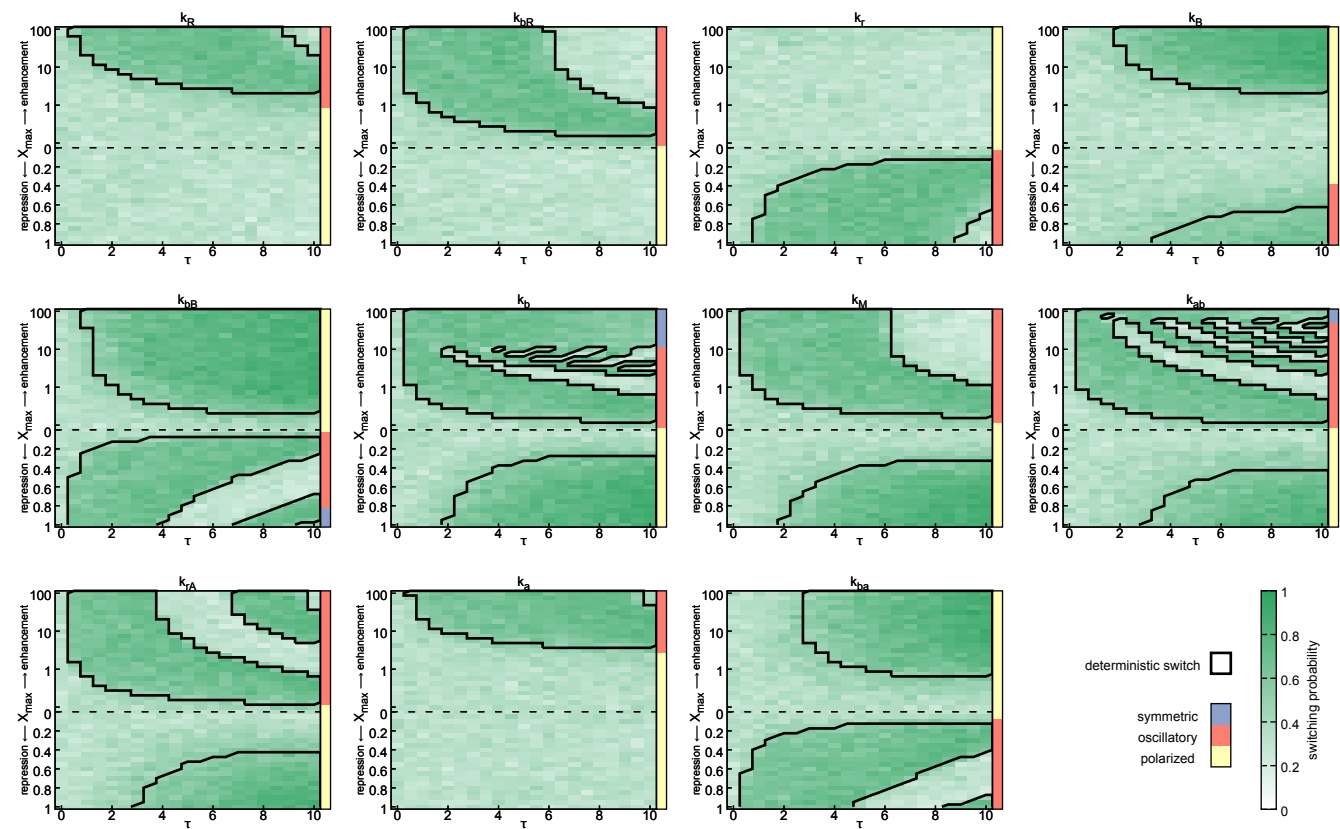

Supplement: S1 Fig — In the shown example, these values were krA = 400 ⋅ 1.01, ka = 2 ⋅ 1.38, kba = 400 ⋅ 0.95, kB = 2 ⋅ 1.15, kbB = 30 ⋅ 0.54, kb = 2.8 ⋅ 0.96, kM = 0.3 ⋅ 1.36, kab = 0.5 ⋅ 30 ⋅ 0.59, kR = 0.1 ⋅ 1.49, kbR = 1.5 ⋅ 0.75, kr = 0.4 ⋅ 0.98, kX = 20 ⋅ 1.16, and kx = 3 ⋅ 0.61. Here, the deterministic switching regimes shift only slightly in the space of signal amplitude and duration, but the sensitivity to noise becomes significantly stronger. However, the qualitative behavior remains the same as in Fig 8 of the main text, with alternating bands and solid regions that show robust deterministic switching as long as the signal amplitude and duration exceed a threshold. (PDF) [file pcbi.1008587.s001.pdf]

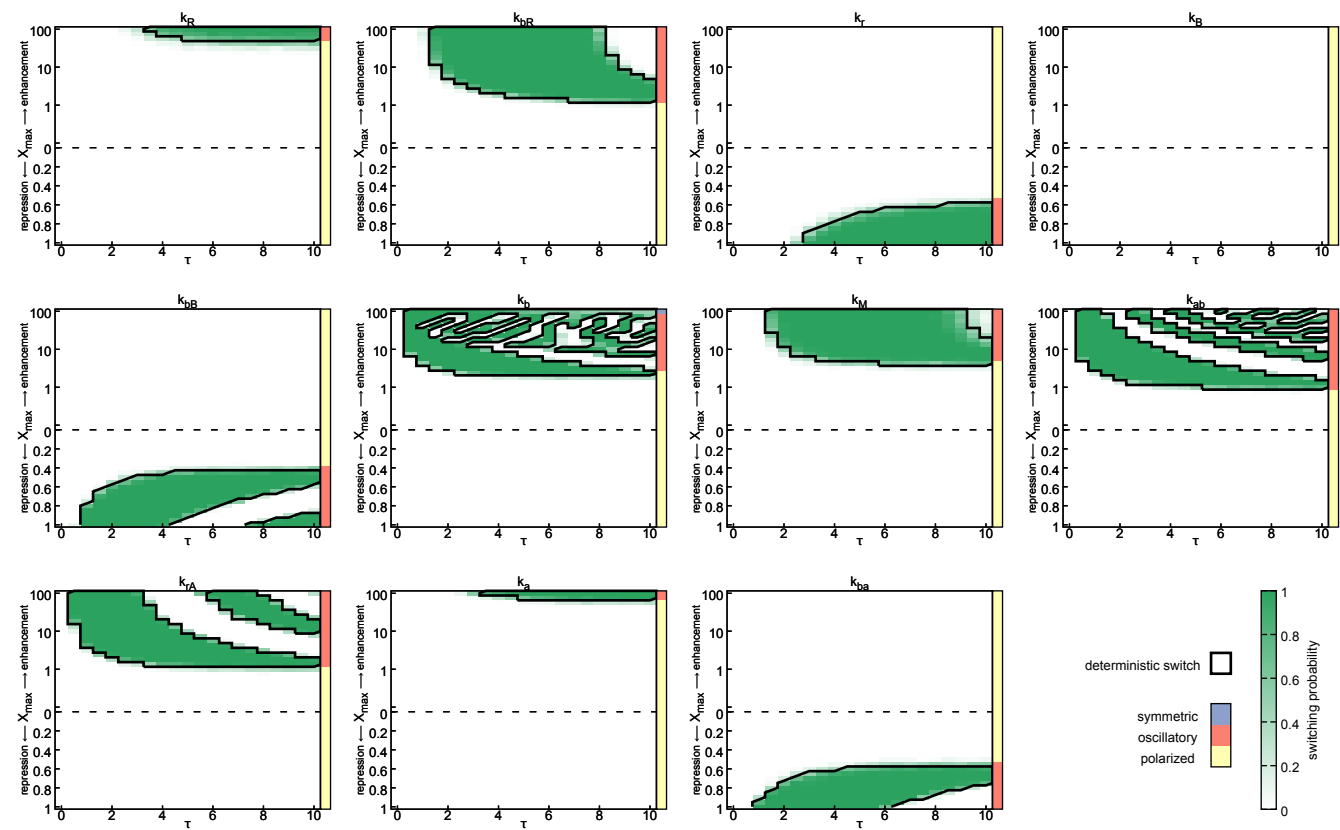

Supplement: S2 Fig — In the shown example, these values were krA = 400 ⋅ 1.1, ka = 2 ⋅ 0.58, kba = 400 ⋅ 1.38, kB = 2 ⋅ 0.81, kbB = 30 ⋅ 1.31, kb = 2.8 ⋅ 0.69, kM = 0.3 ⋅ 1.02, kab = 0.5 ⋅ 30 ⋅ 1.04, kR = 0.1 ⋅ 1.37, kbR = 1.5 ⋅ 1.05, kr = 0.4 ⋅ 1.46, kX = 20 ⋅ 1.08, and kx = 3 ⋅ 1.35. Here, the deterministic switching regimes shift significantly in the space of signal amplitude and duration, and the sensitivity to noise becomes significantly weaker. However, the qualitative behavior remains the same as in Fig 8 of the main text, with alternating bands and solid regions that show robust deterministic switching as long as the signal amplitude and duration exceed a threshold. (PDF) [file pcbi.1008587.s002.pdf]

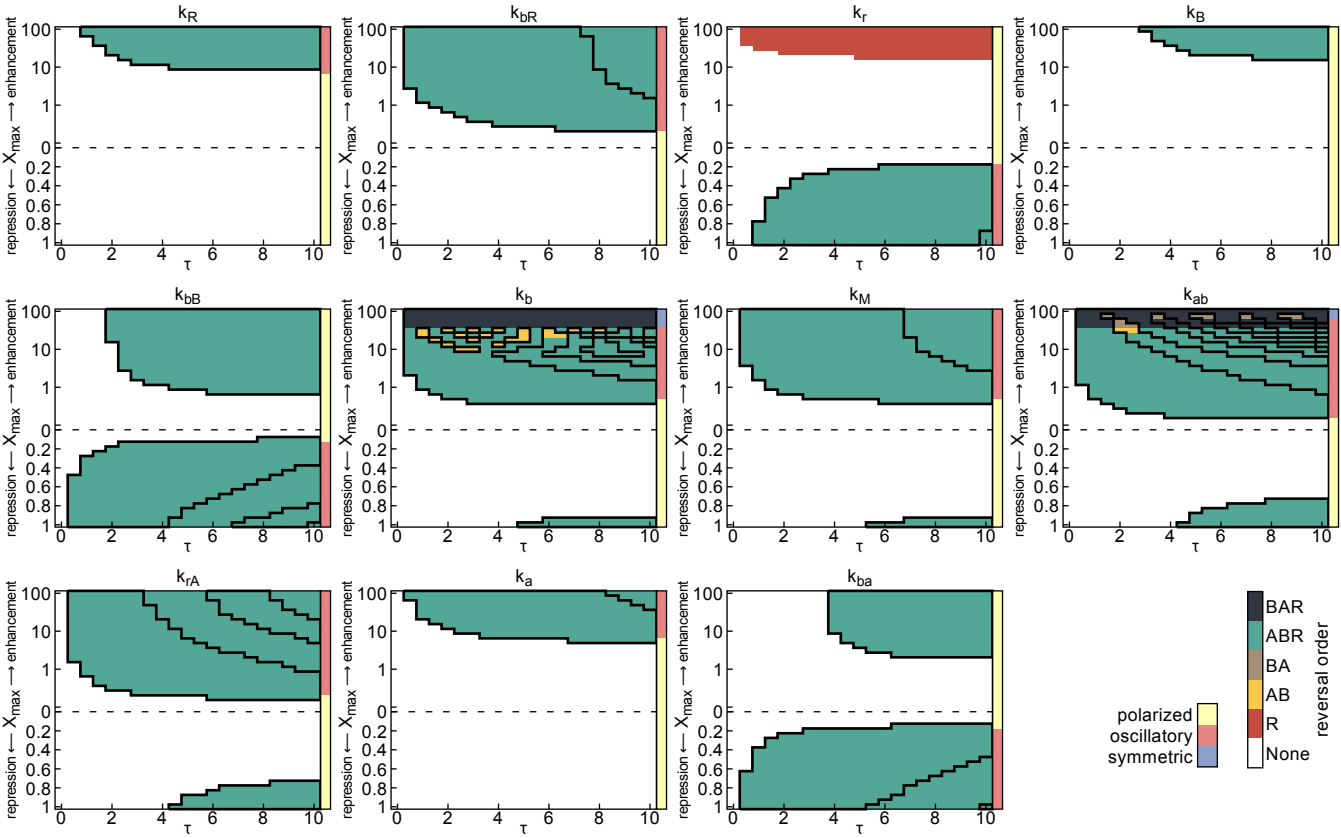

Supplement: S3 Fig — Switching trajectories are obtained from the deterministic model. Black solid lines in the phase diagrams show switching regimes as in Fig 8. The colors indicate in which order A, B and R switch polarity. In the regimes where the system switches polarity multiple times (due to the transient oscillator switch), the switching order represents the order of the first switch. (PDF) [file pcbi.1008587.s003.pdf]

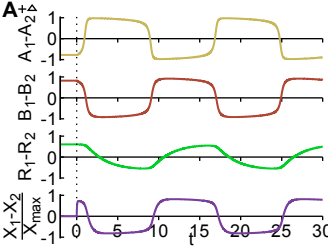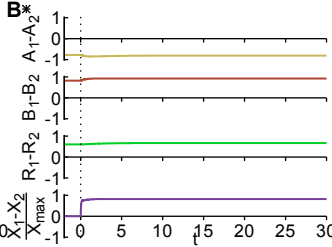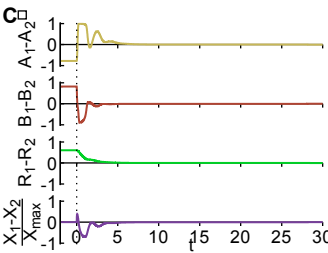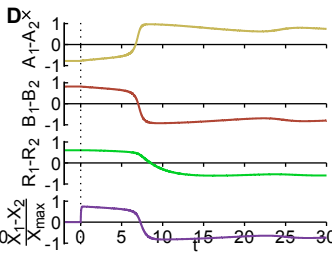

Supplement: S4 Fig — Trajectories of the system during the signal for A the transient oscillator switch, B the prime-release switch, C the Reset switch and D the push switch. The symbols next to the panel labels indicate the signal parameter Xmax as indicated in Fig 8. The signal is applied for the duration of the simulation. During the transient oscillator switch (A) the polarity of the system oscillates, while during the reset switch (C) there is no polarity, i.e. the distribution of the proteins at pole 1 and pole 2 is symmetric. During the prime-release and push switch (B and D) the system is polarized during the switch. (PDF) [file pcbi.1008587.s004.pdf]

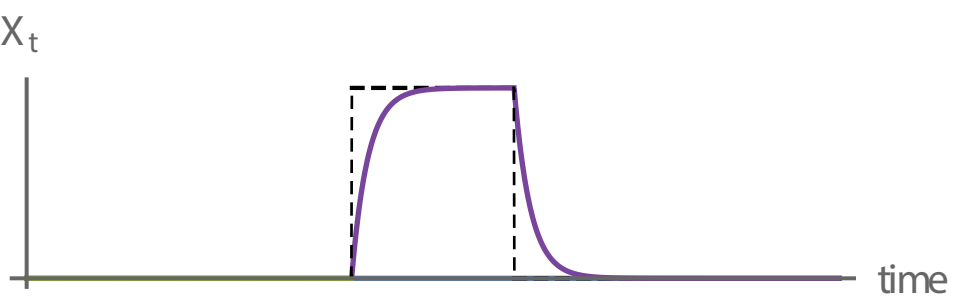

Supplement: S5 Fig — The total amount of X, Xt, increases according to Xt(t) = Xmax(1 − e−λt) for 0 < t < τ and decreases according to Xt(t) = Xmax(1 − e−λτ)e−λ(t − τ) for t > τ. The dashed line indicates the step-like signal. (PDF) [file pcbi.1008587.s005.pdf]

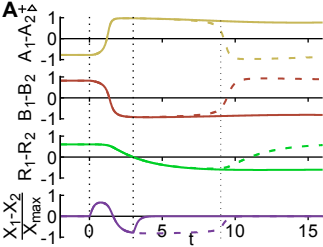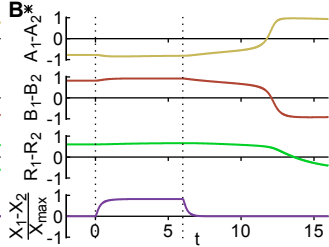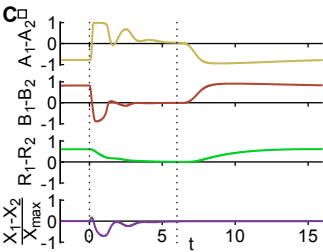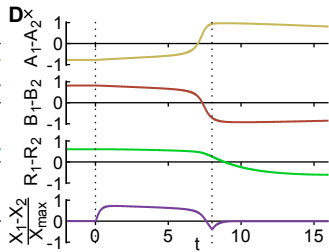

Supplement: S6 Fig — Signal amplitude Xmax and duration τ are chosen the same as in Fig 4, where in A the solid line corresponds to the short signal (plus-symbol) and the dashed line to the long signal (open triangle). The system shows qualitatively the same behavior as for the step-like signal. (PDF) [file pcbi.1008587.s006.pdf]

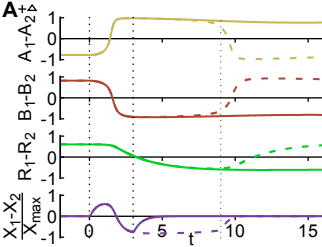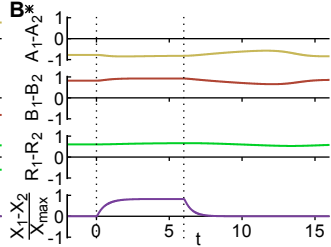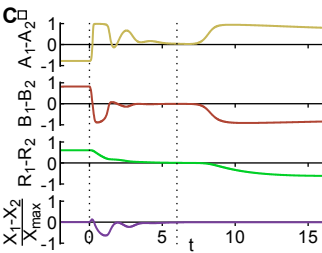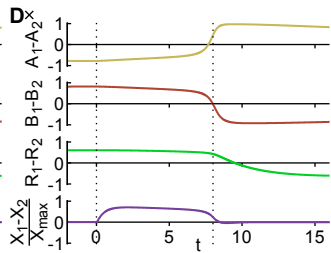

Supplement: S7 Fig — Signal amplitude Xmax and duration τ are chosen the same as in Fig 4, where in A the solid line corresponds to the short signal (plus-symbol) and the dashed line to the long signal (open triangle). For these gradual signals, the transient oscillator switch (A), the reset switch (C) and the push switch (D) switch qualitatively the same as for a step-like signal, while the prime-release switch (B) does not respond to the gradual signal. (PDF) [file pcbi.1008587.s007.pdf]

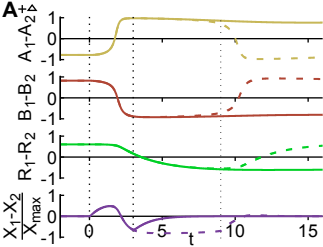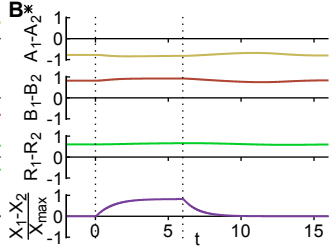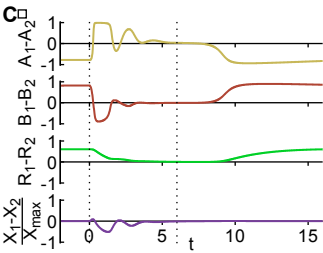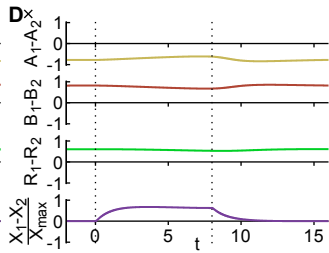

Supplement: S8 Fig — For these gradual signals, the prime-release (B) and push switch (D) do not respond to the signal, while the transient oscillator (A) and reset switch (C) do. (PDF) [file pcbi.1008587.s008.pdf]

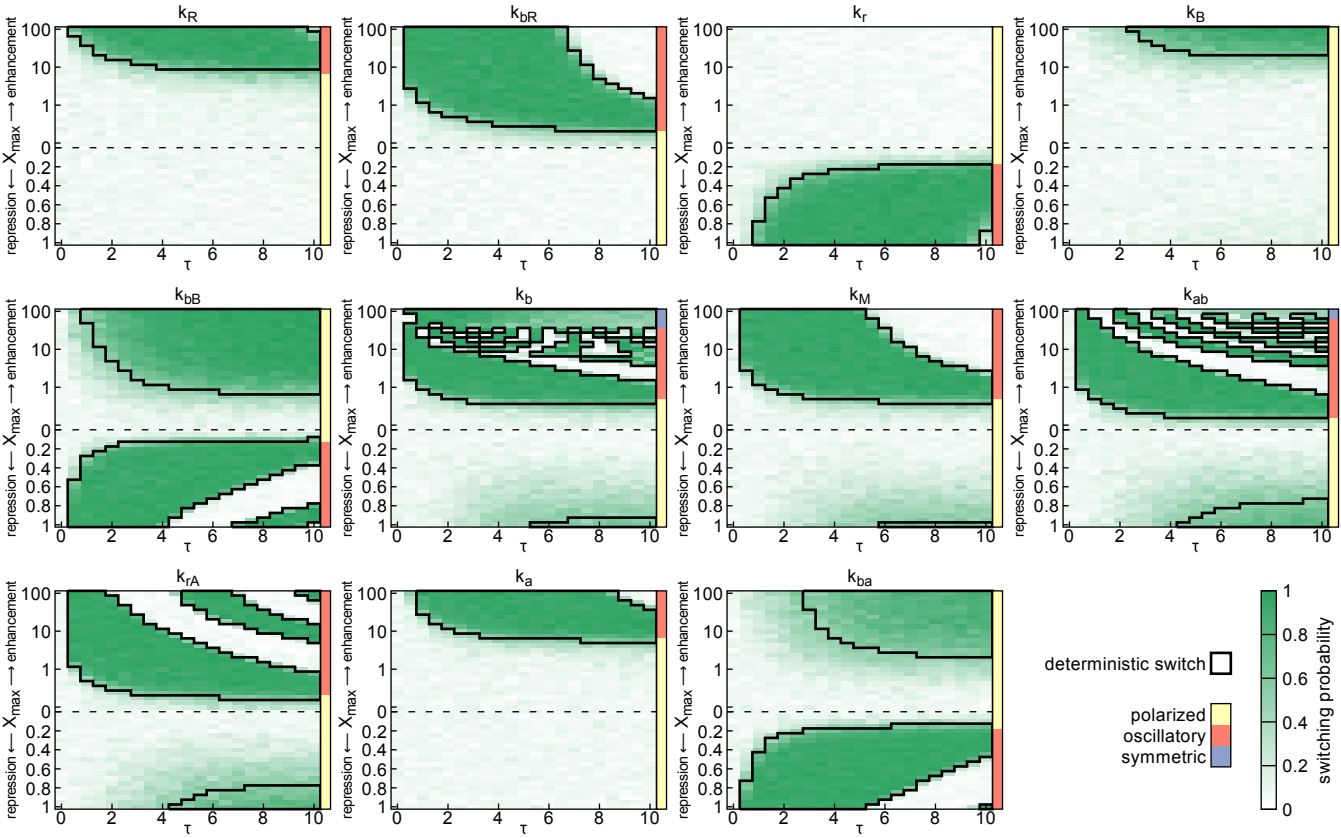

Supplement: S9 Fig — Regions in which the deterministic model shows switches are indicated by thick black outlines. The green shading shows the switching probability of the stochastic model with N = 103.75. The upper half of the phase diagram shows results for a signal that enhances the reaction rate, and the lower half for a repression of the rate. The colored bars to the right of each panel indicate the class of dynamics when the corresponding amplitude of signal is applied, with yellow for polarized, orange for oscillatory and blue for symmetric polar distribution of A, for a gradually increasing and decreasing signal. The switching regimes are similar to the regimes for a step-like signal as shown in Fig 8. (PDF) [file pcbi.1008587.s009.pdf]

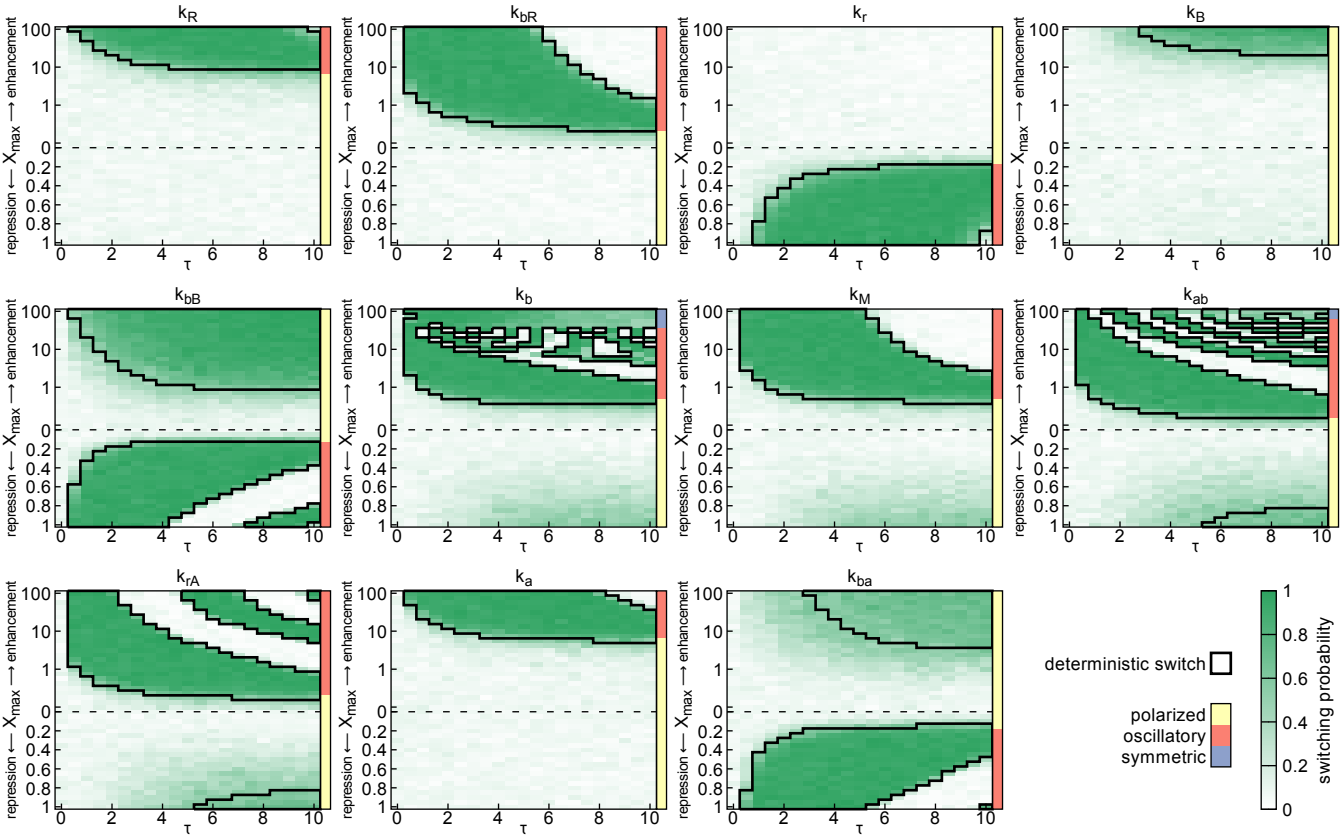

Supplement: S10 Fig — The regimes where the prime-release switch acts to switch the polarity, for example via repression of the parameter kab or krA, have become smaller. (PDF) [file pcbi.1008587.s010.pdf]

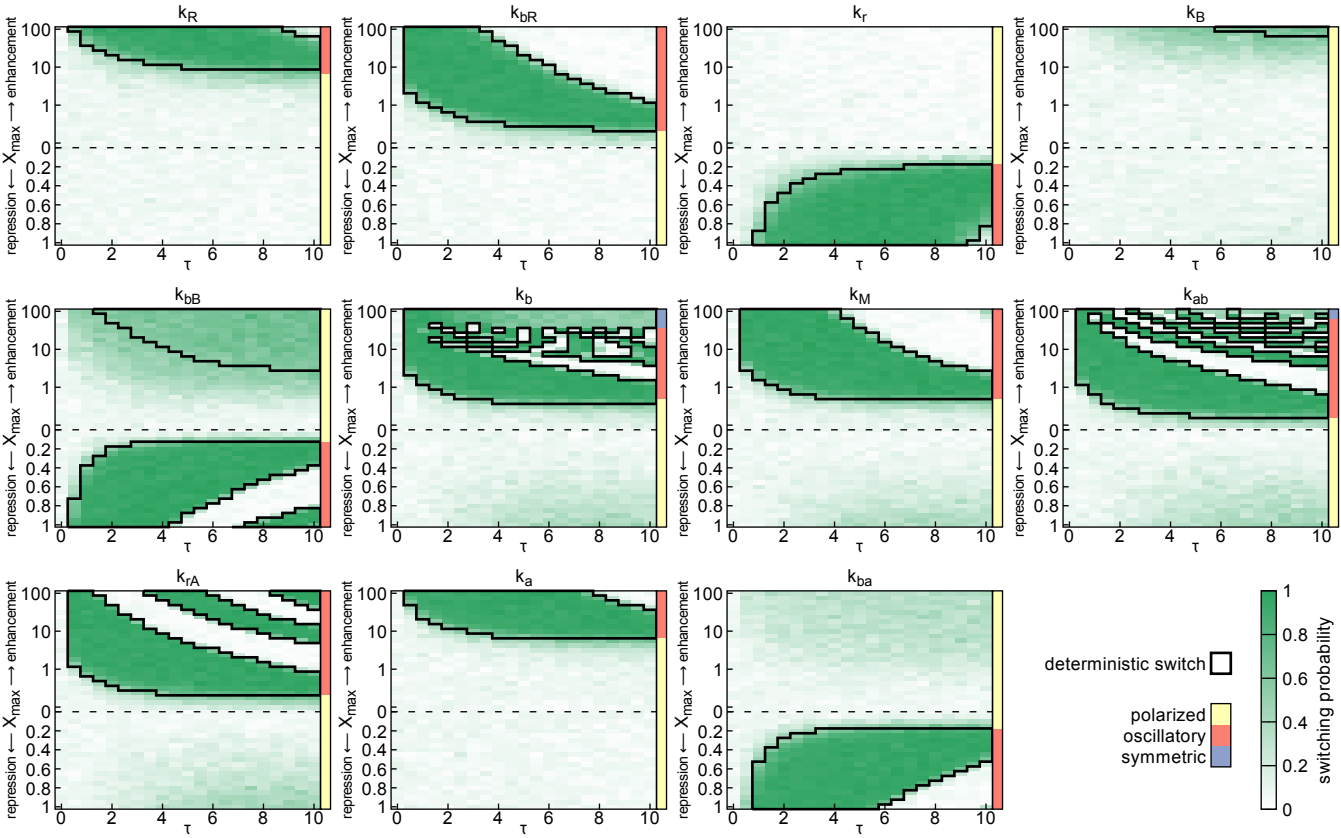

Supplement: S11 Fig — The regimes where the prime-release switch acts to switch the polarity becomes smaller, for example by enhancing kB, or completely vanishes, for example via repression of the parameters kab or krA. In addition, the regimes where the push switch acts vanishes, for example via a slight enhancement of the parameter krA. (PDF) [file pcbi.1008587.s011.pdf]

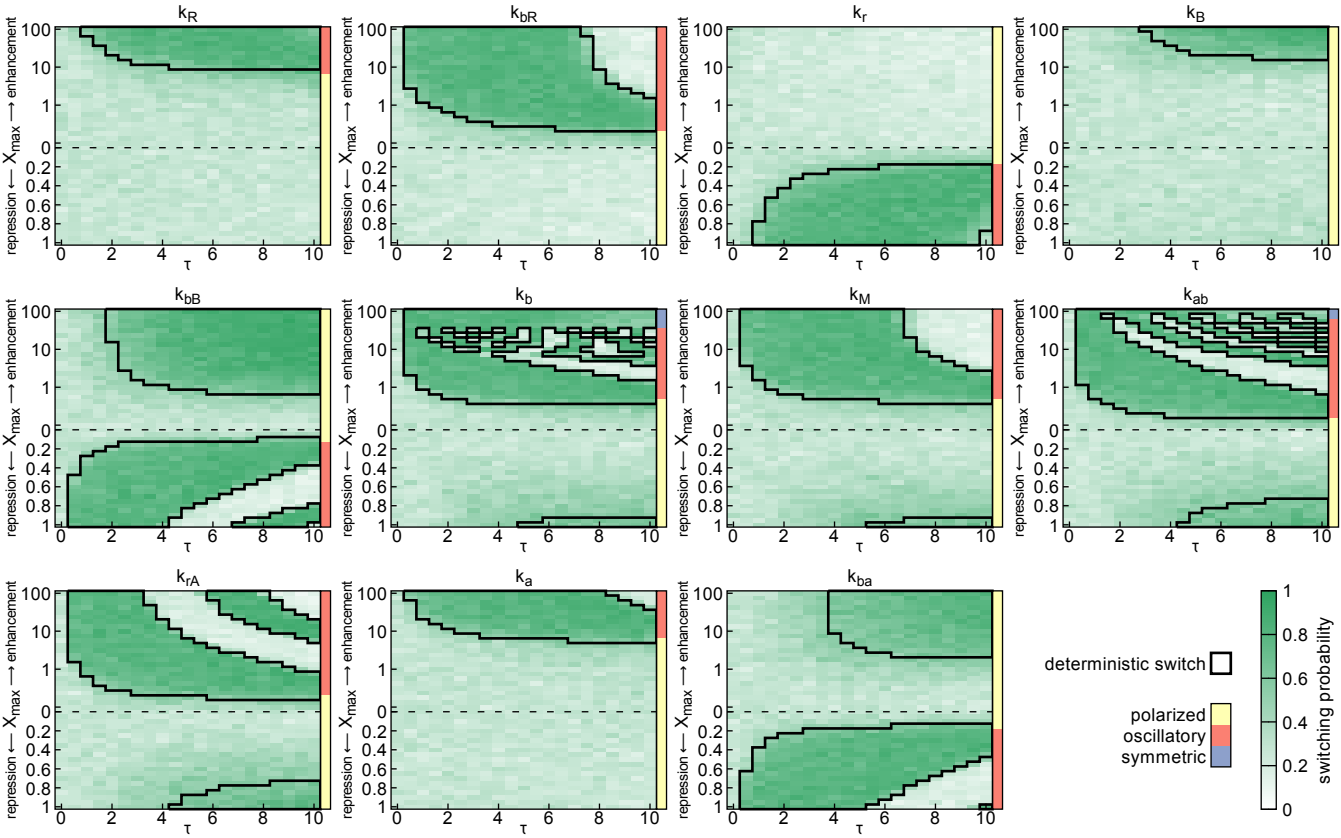

Supplement: S12 Fig — The green shading shows the switching probability of the stochastic model with N = 103.5. The stochastic switching probability, outside of the deterministic switching regimes (solid black lines), is higher as compared to a noise level of N = 103.75 as shown in Fig 8, while the switching probability in the deterministic regimes is smaller. (PDF) [file pcbi.1008587.s012.pdf]

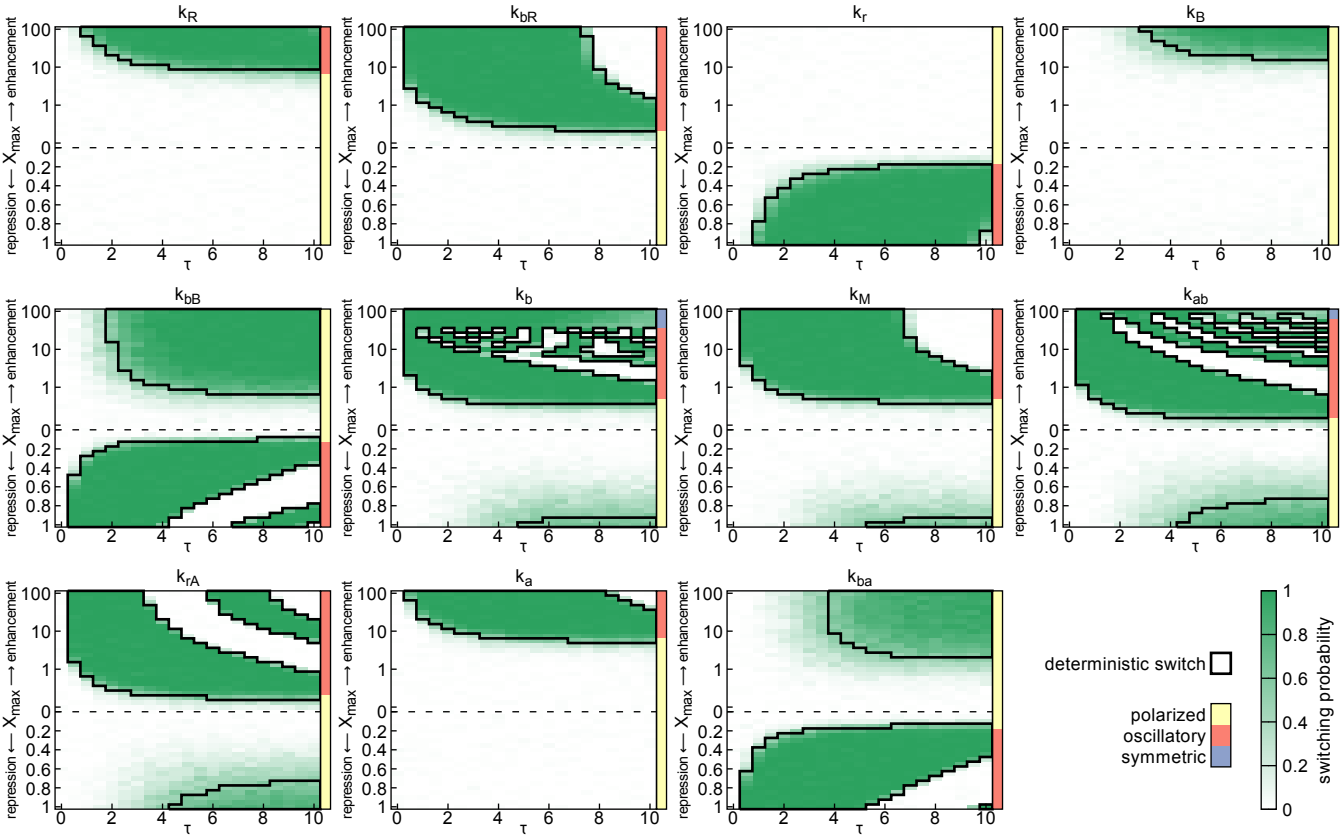

Supplement: S13 Fig — The green shading shows the switching probability of the stochastic model with N = 104. The stochastic switching probability, outside of the deterministic switching regimes (solid black lines), is smaller as compared to a noise level of N = 103.75 as shown in Fig 8, while the switching probability in the deterministic regimes is higher. (PDF) [file pcbi.1008587.s013.pdf]

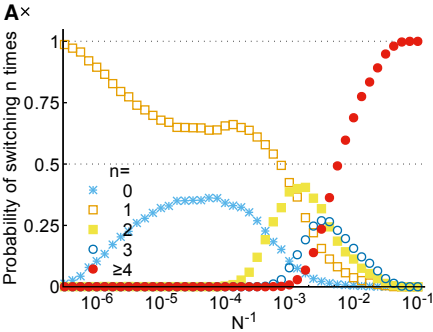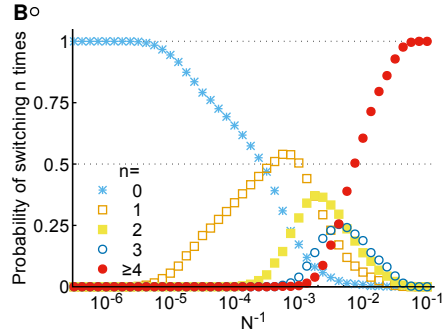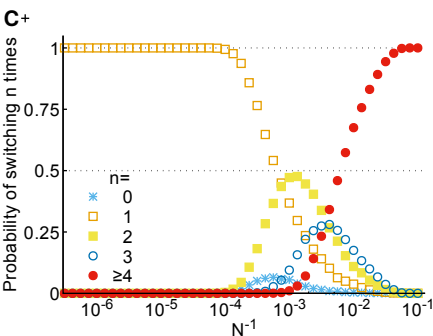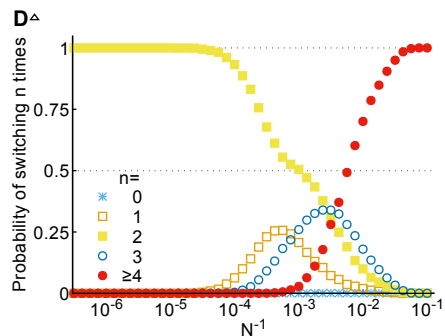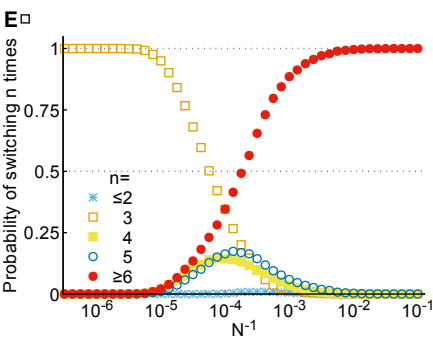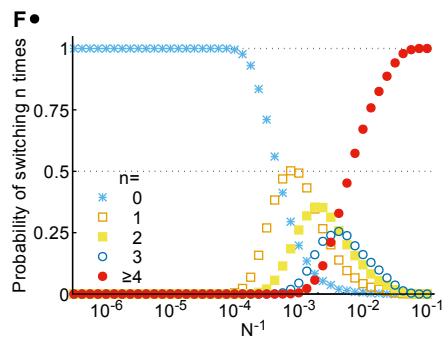

Supplement: S14 Fig — Symbols next to the panel labels A-E correspond to the signal amplitude and duration as shown in Fig 8. F shown the probability of different numbers of switches without a signal. (PDF) [file pcbi.1008587.s014.pdf]

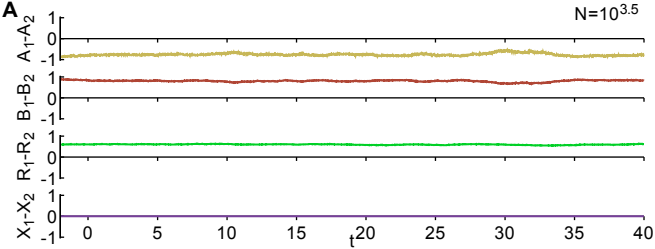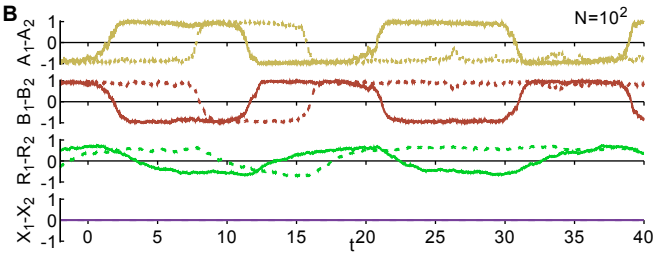

Supplement: S15 Fig — A for low noise levels (N = 103.5) the system does not switch for the duration of the simulation. B for high noise levels (N = 102) the polarity switches several times without applying a signal. (PDF) [file pcbi.1008587.s015.pdf]

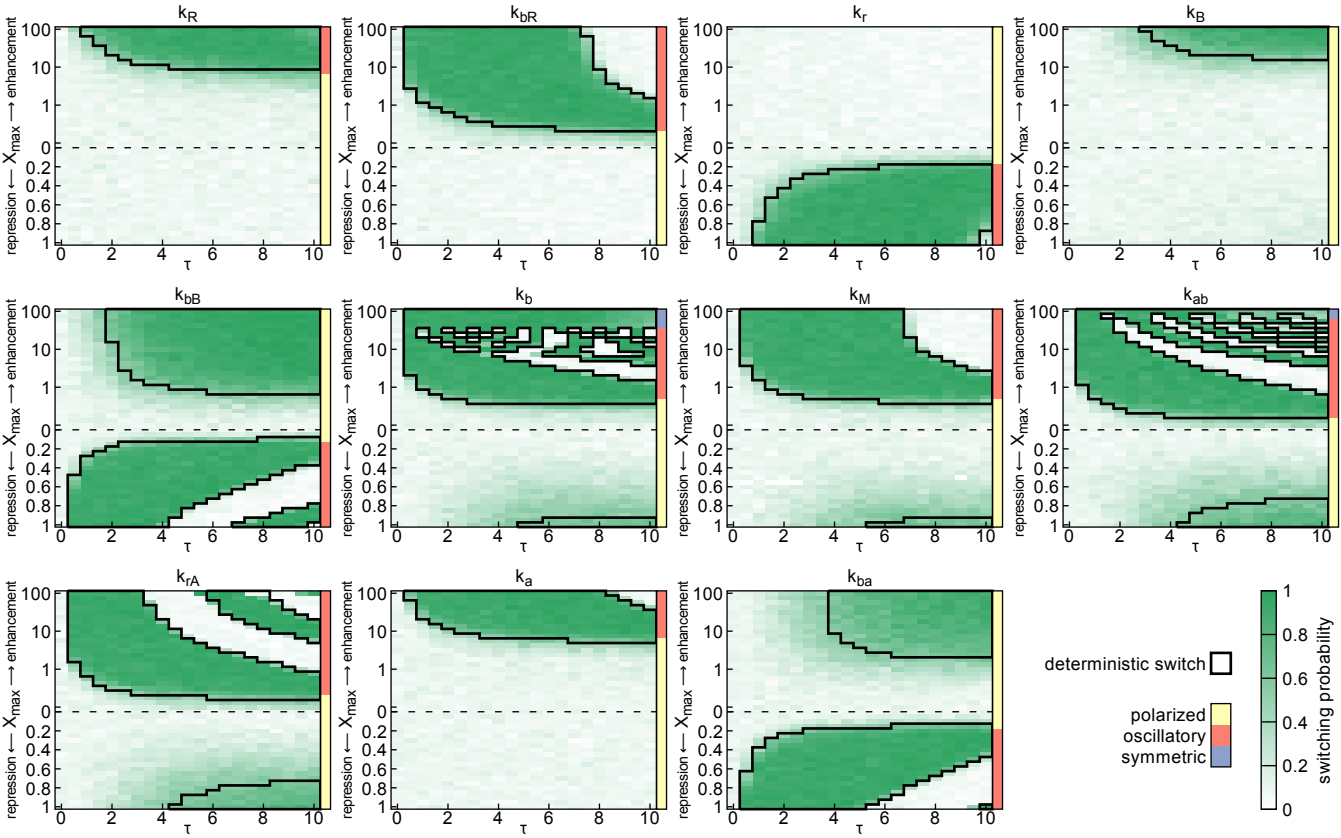

Supplement: S16 Fig — The green shading shows the switching probability of the stochastic model with white noise and with N = 104. The switching regimes are qualitatively similar to the switching regimes in Fig 3. (PDF) [file pcbi.1008587.s016.pdf]

Switching probability

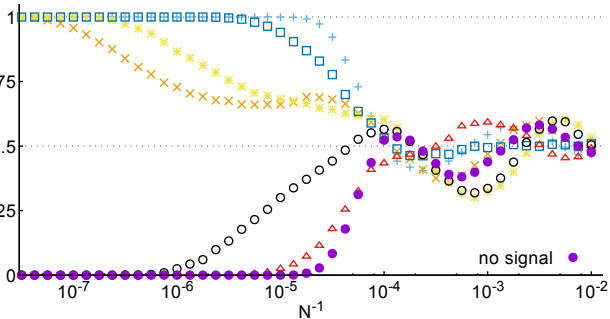

Supplement: S17 Fig — The signal parameters are indicated by the corresponding symbols in Figs 8 and 4. Results are qualitatively similar to the results presented in Fig 6A. (PDF) [file pcbi.1008587.s017.pdf]
