# Supplementary material for: Four different mechanisms for switching cell polarity
Source: PLoS Comput Biol. 2021 Jan 19;17(1):e1008587. doi: 10.1371/journal.pcbi.1008587 (PMC7861558; doi:10.1371/journal.pcbi.1008587)
Supplement: Attachment — Submitted filename: ReviewComments.pdf [file pcbi.1008587.s018.pdf]

Report on "Four different mechanisms for switching cell polarity"

By Filipe Tostevin, Manon Wigbers, Lotte Søgaard-Andersen, and Ulrich Gerland

The authors used a mathematical model consisting of three kinds proteins to explore the switching mechanisms of cell polarity. They conducted simulations in both deterministic and stochastic systems and revealed four kinds of switching mechanisms with different characteristics. In their numerical studies, they demonstrated why the four types of mechanisms behave differently to the signal and the noise. The results are also useful for us to understand other biological switching processes. The paper is well written and interesting to read, so I would recommend it for publication after revision (according to following comments).

The followings are some comments:

1. In the introduction, some related works done by Yi's Lab can be discussed to strengthen the content of the introduction. For example,

   Chou, C.-S., Bardwell, L., Nie, Q. & Yi, T.-M. Noise filtering tradeoffs in spatial gradient sensing and cell polarization response. *BMC Systems Biology* **5**, 196 (2011).

   Lawson, M. J., Drawert, B., Khammash, M., Petzold, L. & Yi, T.-M. Spatial stochastic dynamics enable robust cell polarization. *PLoS Computational Biology* 9, e1003139 (2013).

2. Page 6, the authors can provide the basic structure of the Langevin equations when they discuss about the stochastic models in the main text.
3. The red symbols in Fig. 3, whose trajectories were later shown in Fig. 4, mainly lie in the eighth sub-figure interpreting the model parameter k_{ab}. Is there any reason for choosing these red points from this sub-figure? Or does it influence the observations or conclusions if I choose these symbols from the other sub-figure, such as the one interpreting the model parameter k_b?
4. Page 7, in Eqs. 4 and 5, index i in X_i is clearly chosen from 1 or 2, but it is however confusing to justify the index j in k_j. It could be better for understanding to give an explanation of the k_j.
5. Page 8, in Fig. 2C, the notation of switching sign \omega_A(0) and \omega_A(t_{end}) is not consistent with it in the caption of Fig. 2.
6. Page 9, the title "Characterizing succesful switching scenarios" should be "Characterizing successful switching scenarios". And this typo also appeared in the paragraph below Fig. 4 on page 11 and in the Fig. 2D on page 8.
7. Page 14, in Fig. 5, what the axes of the figures represent are not clearly explained in the caption and the main content.

8. Page 14, in Fig. 5 caption, the sentence "……black lines when the signal is switches off." should be "…… is switched off."; Page 19, in the sentence "for the system to cross into the opposite basin of attraction requires……", the "for" should be deleted; Page 21, the word "phenomonology" should be "phenomenology"; Page 24, in the last paragraph, there is "…… they fell of the separatrix……" and "The projections……was then estimated…".
9. Page 16, in Fig. 6, does "n" represent the number of switching events? It seems that the explanation is not included in the content.
