# Supplementary material for: Four different mechanisms for switching cell polarity
Source: PLoS Comput Biol. 2021 Jan 19;17(1):e1008587. doi: 10.1371/journal.pcbi.1008587 (PMC7861558; doi:10.1371/journal.pcbi.1008587)
Supplement: Attachment — Submitted filename: Response to the Reviewers.pdf [file pcbi.1008587.s019.pdf]

Response to the Reviewers' comments

We thank the Reviewers for their time and effort in evaluating our manuscript, and for their helpful comments. In the following, we quote the Reviewers' comments (in blue italic font) and supply a point-by-point response.

Peer Reviewer #1 Evaluations:

The authors used a mathematical model consisting of three kinds proteins to explore the switching mechanisms of cell polarity. They conducted simulations in both deterministic and stochastic systems and revealed four kinds of switching mechanisms with different characteristics. In their numerical studies, they demonstrated why the four types of mechanisms behave differently to the signal and the noise. The results are also useful for us to understand other biological switching processes. The paper is well written and interesting to read, so I would recommend it for publication after revision (according to following comments).

We thank the Reviewer for the appreciation of our work. We have carefully addressed all questions and comments, as detailed in our point-by-point response below.

The followings are some comments:

1. In the introduction, some related works done by Yi's Lab can be discussed to strengthen the content of the introduction. For example,

Chou, C.-S., Bardwell, L., Nie, Q. & Yi, T.-M. Noise filtering tradeoffs in spatial gradient sensing and cell polarization response. BMC Systems Biology 5, 196 (2011).

Lawson, M. J., Drawert, B., Khammash, M., Petzold, L. & Yi, T.-M. Spatial stochastic dynamics enable robust cell polarization. PLoS Computational Biology 9, e1003139 (2013).

We thank the Reviewer for pointing us to these references. In the revised version of our manuscript, we cite both papers in the introduction, to highlight the role of noise in cell polarization.

2. Page 6, the authors can provide the basic structure of the Langevin equations when they discuss about the stochastic models in the main text.

In the revised manuscript, we now describe the basic structure of the Langevin equations in one sentence, and now also directly refer to the equation number in the Materials and Methods section.

3. The red symbols in Fig. 3, whose trajectories were later shown in Fig. 4, mainly lie in the eighth sub-figure interpreting the model parameter k_{ab}. Is there any reason for

choosing these red points from this sub-figure? Or does it influence the observations or conclusions if I choose these symbols from the other sub-figure, such as the one interpreting the model parameter k_b?

Our reasoning for choosing several examples from the same sub-figure was to illustrate that even with the same regulatory network, one can get different types of switching mechanisms depending only on the signal amplitude, duration, and sign of regulation (enhancing or inhibitory). To make this clearer, we added the following sentence in the beginning of the Discussion section:

“Interestingly, one can obtain different switching mechanisms already with the same signaling and regulation network, by changing only the signal amplitude and duration, or the sign of the regulatory effect of the signal (Fig. 3).”

Otherwise the choice does not matter, i.e. the qualitative dynamics for a given type of switch is the same, regardless of the specific regulatory network and parameters. The case of k_{ab} is also interesting, because the Guzzo et al paper posits that the signal acts through this parameter.

4. Page 7, in Eqs. 4 and 5, index i in X_i is clearly chosen from 1 or 2, but it is however confusing to justify the index j in k_j. It could be better for understanding to give an explanation of the k_j.

We thank the Reviewer for pointing out this possible obscurity. In the revised manuscript, we now explain this more clearly by writing:

“In each case, one parameter, denoted k_j, depends on X_i while the others are not affected (the index j in k_j specifies which of the 11 parameters in Eqs. 2 is regulated).”

5. Page 8, in Fig. 2C, the notation of switching sign \omega_A(0) and \omega_A(t_{end}) is not consistent with it in the caption of Fig. 2.

We thank the Reviewer for pointing us to this inconsistency, which is now corrected.

6. Page 9, the title “Characterizing succesful switching scenarios” should be “Characterizing successful switching scenarios”. And this typo also appeared in the paragraph below Fig. 4 on page 11 and in the Fig. 2D on page 8.

We corrected all of these typos.

7. Page 14, in Fig. 5, what the axes of the figures represent are not clearly explained in the caption and the main content.

Prompted by a comment of Reviewer #2, we replaced the schematics that were previously shown in Fig. 5 by actual phase space projections, which are now shown in the new figures 5 and 6 (due to the size of these new figures, the old panel A is split into

a separate figure, which is the new Fig. 5, and the old panels B-E have now become the new Fig. 6). The meaning of the axes in these new figures is described.

8. Page 14, in Fig. 5 caption, the sentence "......black lines when the signal is switches off." should be "...... is switched off."; Page 19, in the sentence "for the system to cross into the opposite basin of attraction requires......", the "for" should be deleted; Page 21, the word "phenomonology" should be "phenomenology"; Page 24, in the last paragraph, there is "...... they fell of the separatrix......" and "The projections......was then estimated...".

We thank the Reviewer for pointing out these typos, which are now fixed.

9. Page 16, in Fig. 6, does "n" represent the number of switching events? It seems that the explanation is not included in the content.

Yes, "n" represents the number of switching events. This was only defined in the y-axis of the plot – now it is properly explained in the caption.

We thank the Reviewer for the careful reading and the helpful comments.

Peer Reviewer #2 Evaluations:

In the paper "Four different mechanisms for switching cell polarity" by Tostevin, Wigbers, Sogaard-Andersen and Gerland, the authors start with a minimal model of cell polarity oscillations, and then systematically explore the effects of different pulse inputs into the model. Through exhaustive simulations they uncover four classes of polarity switching behaviors. They characterize each switching mechanism including the effects of stochasticity by showing the switching regimes, and state space behaviors. Introducing stochastic effects increases the probability of switching including noise-driven oscillatory switching (coherence resonance).

It is surprising that the model was able to generate four distinctly different dynamical behaviors based on how the input was introduced into the model. The modeling approach was clever, introducing the input at each parameter with systematic changes in signal amplitude and length. The biological relevance of these different behaviors is less clear.

I believe that the authors have achieved a solid understanding of the novel system behaviors, but that the characterization needs more support in certain areas for a more complete story. Overall, there are interesting innovations in both approach and results.

The authors conclude that they have "developed a general classification of signal-induced polarity switching mechanisms based only on how a signaling protein changes the topology of the polarity system's state space". I quibble with the generality of the findings, but their treatment is broader than currently exists.

My recommendation is for a revise and resubmit with certain sections strengthened to maximize impact. My primary recommendations are listed below; more minor edits mainly focused on presentation will be provided in the next review cycle.

We thank the Reviewer for this concise assessment and the appreciation of our work. We have carefully addressed all questions and comments, as detailed in our point-by-point response below.

1. Figure 5 which is a schematic figure in the middle of the Results section needs to be replaced by phase plane diagrams generated from actual data along the lines of what is done in Figure 6D.

Our motivation for showing the schematic Fig. 5 in the middle of the Results section was to make it easier for the reader to understand the basic working principles of the four types of switches, and the differences between them. The actual phase plane projections are relatively cumbersome to interpret, such that a casual reader may have difficulty to extract the underlying working principles. These working principles are part of our core message, and therefore important to communicate effectively. That being said, the point of the Reviewer is well taken, since the schematic figure communicates the result, but does not support it by direct evidence.

During the revision process, we spent a significant amount of time considering how to present both, the direct evidence and the conclusion, in a clear but reasonably concise way. We ultimately converged to a solution that requires a bit more space (the total figure count is increased by two), but satisfies all criteria. In the Results section, we replaced the old schematic Fig. 5 by two figures that show actual data. The new Fig. 5 replaces the old Fig. 5A. It displays the three qualitatively different dynamics that can occur in the presence of the signal, by showing trajectories and invariant sets (i.e., fixed points and limit cycles) in the three-dimensional state space (A_1-A_2, B_1-B_2, R_1-R_2). The new Fig. 6 displays evidence for the four different working principles, by showing, for each of the four types, the dynamics of signal-induced switching in three different state space projections. Here, insets magnify important features of these trajectories as needed. Together, the new Figs. 5 and 6 supply the direct evidence for the conclusions drawn in the Results section.

However, we did not want to leave it at that: We also included an adapted version of the old, schematic Fig. 5B-E in the Discussion section, for two reasons. First, we wanted to graphically summarize the working principles of the four switching mechanisms. Second, this figure is helpful for the discussion of the generality (and its limitations) of our classification of switching mechanisms. These aspects are now discussed in much more detail in the revised Discussion section, to address point #5 of the Reviewer, as described below.

2. Exploration of parameter space. A key question is whether these findings are valid only for one particular parameter set (one point in parameter space) or apply broadly to a wide range of parameter values in the model. The authors need to explore parameter space, not necessarily in an exhaustive fashion but in a targeted fashion and compare the results from different parameter points in an additional figure (which can be placed in the supplement). This would address the critique whether the results are robust to parameter variation.

We followed this request of the Reviewer and repeated the analysis shown in Fig. 3 for two other parameter sets, i.e. two other points in parameter space, which were randomly chosen (by multiplying each of the original parameter values by a random number between 0.5 and 1.5). The resulting two new figures analogous to Fig. 3 are shown in the supplement. It can be seen that the deterministic switching regimes shift in the signal amplitude/duration space, and the sensitivity to noise can become stronger or weaker. However, the qualitative behavior remains the same, in particular the existence of `alternating bands' (representing transient oscillator switches) and the solid regions that show robust deterministic switching as long as the signal amplitude and duration exceed a threshold.

The fact that the qualitative deterministic dynamics are insensitive to the parameter values (within a certain regime) is also consistent with nonlinear dynamical systems theory: To change the qualitative behavior, a bifurcation must occur, which requires that the system is moved through a lower-dimensional surface in parameter space. Hence,

unless the original point in parameter space is exactly on such a lower-dimensional bifurcation surface (which has measure zero in the total parameter space), there must be an open set of finite volume around the original point, where the system dynamics is qualitatively the same. This generic argument clearly cannot say anything about the size of that volume. Our numerical experiment shows that it is not tiny, which we take is what the Reviewer would like us to establish here.

3. What is the biological significance of the work? In other words why is this paper in PLoS Computational Biology and not a SIAM or a Physics journal? I would like the authors to focus on two areas in the Discussion. First what are the implications regarding the possible function of FrzX. The original model has X affecting the k_{ab} term. Are some of the other simulations consistent with the data in the original paper suggesting other alternative roles for FrzX? Second, are there examples of the various spatial switching dynamics in other biological pattern forming systems? If not, the authors should say clearly that their results mainly apply to the M. xanthus system (at least for now). These points can be addressed in the Discussion.

We agree with the Reviewer that an expanded discussion of these questions benefits the paper. Currently, the *M. xanthus* system is arguably the best studied system for polarity switching, but even there it is not yet settled what the actual switching mechanism is. The paper by Guzzo et al (Nat. Microbiol, 2018) made a big step forward by identifying the new component FrzX and proposing one possible mechanism for the observed polarity switching, but other possible mechanisms are currently not excluded. Furthermore, important new components of this system continue to be found (e.g., PMID: 31110363) and some of the detailed model assumptions in the Guzzo et al study appear inconsistent with more recent data (PMID: 32569324). So, the situation remains in flux regarding *M. xanthus*, and this is even more true for other experimentally studied examples of polarity switching, such as neutrophils, which we discussed in the introduction of our manuscript.

Given this state of research, the biological significance of our work is to “put the options on the table”, i.e. to clearly state which types of mechanisms are in principle available, and what the properties of these mechanisms are. To clearly distinguish between these mechanisms, it will be important to have experimental control over the input signal that triggers polarity switching. This is now discussed more extensively in the revised Discussion section. Unfortunately, it is currently not possible to experimentally control the input signal for the *M. xanthus* system, partly because the actual input signal has not yet been identified. In the revised Discussion section, we now also explain the more general biological significance of this work, as a guide for future experiments probing the mechanisms of signal-induced polarity switching in model systems ranging from bacteria to neutrophils.

4. What is the effect of diffusion? Again this point can be addressed in the Discussion (as a possible future direction). The model makes a simplification (which must be noted in the main text) that the interior of the cell is well-mixed. Would the results change if a time delay via diffusion is introduced as the species traverse from one pole to the other?

This is an important question, which we had only briefly touched upon in the second paragraph of the Results section. The simplification made by our model is essentially an assumption of time-scale separation, between the diffusive transport and the relevant biochemical processes. Given typical cell lengths (e.g. L~6 μm for *M. xanthus*) and diffusion coefficients for a small cytoplasmic protein (e.g. D~10 μm^2/s, see Mullineaux et al, J Bacteriol., 2006), the mixing timescale $L^2/(2D)$ over which cytoplasmic proteins explore the bulk of the cell is less than 2 seconds. In contrast, the observed timescale of the actual switching process, during which the abundance of the polarity system proteins decrease at the one pole and increases at the opposite pole, is on the order of 30 seconds in the case of *M. xanthus* (Fig. 1 in Guzzo et al, Nat. Microbiol, 2018). This, suggests that the biochemical processes set the time-scale of the dynamics rather than the diffusive transport, such that it is reasonable to approximate the cytoplasm as being well-mixed. However, it will certainly be interesting to explore the dynamics under conditions where this approximation no longer holds. In the revised manuscript, we now mention this in the Discussion section as a possible future direction.

5. The following claim in the Discussion is not supported by any argument: "From a nonlinear dynamics perspective, the four types of behavior in Fig. 5B-E appear to exhaust the spectrum of possible behaviors, such that we do not expect additional classes to appear in other polarity models." This sentence should be removed or supported by data.

We thank the Reviewer for pushing us to clarify and substantiate this statement. We rewrote this part of the Discussion section, making use of the old, schematic Fig. 5B-E to illustrate our reasoning (new Fig. 9 in the revised Discussion section). The starting point of this reasoning is the phase space structure of a stable polarity system, i.e. without the signal input that triggers polarity switching. Irrespective of how this (binary) polarity system is realized, i.e. how many components it has and how they interact, the phase space structure must be that of a bistable system. In other words, given a D-dimensional phase space (where D depends on the number of components), we must have two stable fixed points with basins of attraction separated by a (D-1)-dimensional separatrix. The system then sits in one of these fixed points with a stable polarity (shown as black filled circles in the two-dimensional illustration of Fig. 9).

When the signaling system is activated, it interferes with the polarity system. This will temporarily "warp" the structure of the phase space, and cause the state of the system to move within the phase space. The movement begins during the application of the signal (red trajectories in Fig. 9), but continues after the signal has disappeared and the structure of the phase space has returned to its original state (black trajectories in Fig. 9).

We found three types of phase space structure in the presence of the signal: monostable, bistable, and oscillatory (Fig. 5). Therefore our classification of signal-induced polarity switches includes only these three basic structures. In principle, there could be polarity networks for which the signaling system induces more complex types

of phase space structure, e.g. higher-order multistable or chaotic, albeit the functional benefits would be unclear. With the three basic structures, our analysis revealed four types of polarity switches. All four have in common that the temporary deformation of the polarity system's phase space structure leaves the system on the other side of the separatrix when the original bistable phase space structure is restored. The transient oscillator switch achieves this by moving the system along a limit cycle during the signal (Fig. 9A), while the reset switch moves it towards a single stable fixed point along a curved trajectory (Fig. 9B). When the system is bistable in the presence of the signal, there are two distinct types of switches: Either the signal moves the fixed point through the original separatrix (prime-release switch, Fig. 9C), or the signal pushes the separatrix through the original fixed point (push switch, Fig. 9D). Based on the geometrical theory of nonlinear dynamical systems, these four types of behavior appear to exhaust the spectrum of possible behaviors. Hence, we do not expect additional classes of signal-induced polarity switches to arise in other models of binary polarity systems, as long as these are either monostable, bistable, or oscillatory in the presence of the signal.

6. I have a small number of minor edits regarding presentation. I will include these in the next review cycle.

We appreciate the well-taken comments of the Reviewer and will be happy to implement any further input to polish this manuscript.
